# I Feel what You Feel if You Are Similar to Me

**DOI:** 10.1371/journal.pone.0004930

**Published:** 2009-03-18

**Authors:** Andrea Serino, Giulia Giovagnoli, Elisabetta Làdavas

**Affiliations:** 1 Dipartimento di Psicologia, Università degli Studi di Bologna, Bologna, Italy; 2 Centro studi e ricerche in Neuroscienze Cognitive, Polo Scientifico-Didattico Cesena, Bologna, Italy; Università di Parma, Italy

## Abstract

Social interactions are influenced by the perception of others as similar or dissimilar to the self. Such judgements could depend on physical and semantic characteristics, such as membership in an ethnic or political group. In the present study we tested whether social representations of the self and of others could affect the perception of touch. To this aim, we assessed tactile perception on the face when subjects observed a face being touched by fingers. In different conditions we manipulated the identity of the shown face. In a first experiment, Caucasian and Maghrebian participants viewed a face belonging either to their own or to a different ethnic group; in a second experiment, Liberal and Conservative politically active participants viewed faces of politicians belonging to their own or to the opposite political party. The results showed that viewing a touched face most strongly enhanced the perception of touch on the observer's face when the observed face belonged to his/her own ethnic or political group.

## Introduction

Vision can influence tactile processing [1] and, in particular, visual information pertaining to the body seems specially effective in modulating the perception of touch [2]. Viewing the body while perceiving touch improves tactile acuity, even when the visual stimulus conveys no information about the tactile stimulus [3]. Several results indicate that the enhancement of tactile perception due to the vision of the body acts at the level of the primary somatosensory cortex. Viewing the body enhanced sensory evoked activity in SI in EEG [4], and MEG [5] studies. Moreover, TMS over SI abolished the beneficial effect of vision on touch [6]. Finally, this effect is ruled by a somatotopic gradient reflecting SI organization [7]: for instance, viewing touch on a part of the body, such as the hand, enhanced somatosensory processing for the viewed body part and for body parts contiguously represented in the SI homunculus, such as the face.

Visual enhancement of touch suggests an important functional relationship between tactile perception and the mental representation of the body as driven by visual information. Similar evidence has shown that tactile perception is influenced not only by visual information about the body, but also specifically by visual information about *touch on a part of the body*. Neuroimaging studies showed that observation of a body being touched evokes brain activity in primary and secondary somatosensory cortices, as well as in some portions of frontal and parietal cortex, even if the observer's body is not directly tactilely stimulated [8]–[10]. This activity does not normally have a perceptual counterpart, as most subjects do not report tactile perception when observing touch on the body of others. An interesting exception is the case of some synesthetic subjects (i.e., visuo-tactile synesthetes), who experience tactile sensation when they see other people's bodies being touched [9], [11]. Notably, in synesthetes, brain activity induced by observation of touch is greater than in non-synesthetes [9]. This finding suggests that a modulation of tactile processing due to vision of touch occurs both in synesthetes and in non-synesthetes, the difference between these groups might be only that sensitivity to the effect is stronger in synesthetes. Coherently with this interpretation, a recent study by our group showed that an effect on tactile perception due to the observation of touch can be unmasked also in non-synesthetic individuals, if tactile stimuli were processed near the perceptual threshold: observing a face being touched by fingers enhanced the detection of around-threshold tactile stimuli on the observer's face [12]. It is worth noting indeed that visuo-tactile integration is maximum when tactile information *per se* is not sufficient to drive a clear percept [13]–[15].

Serino et al. [12] also showed that this *visual remapping of touch* was maximum, when subjects observed their own face being touched instead of the face of others. This finding suggests that visual remapping of touch increases if the observer's body and the observed body matches. In order to re-map a sensation from one sensory modality, namely vision, to another sensory modality, namely touch, the remapping is probably favoured if the two modalities share a common reference system, that is, the same body. Thus, visual remapping of touch depends not only on visual information about *touch* but also on visual information about the body.

Body-related visual information provides both *physical* and *semantic* cues about the self and co-specifics: this information allows the observer to categorize others as either similar to oneself, i.e. in-group, or dissimilar, i.e. out-group. In the present study, we asked whether physical or semantic similarity between the self and others is incorporated into the mental representation of the body, thereby affecting visuo-tactile re-mapping. A basic form of categorization of others as similar or dissimilar to the self is based on physical traits defining the membership to a given ethnic group. To study whether this effect modulates visual remapping of touch, in Experiment 1, 7 Caucasian and 7 Maghrebian subjects were asked to observe a picture of a face being touched by hands while receiving sub-threshold tactile stimuli on their face. The observed face could belong to the same ethnic group as the observer (In-group) or to the other ethnic group (Outgroup). If visual remapping of touch is modulated by the *physical similarity* between the observer and the observed face, then tactile perception should be enhanced when people observe touch directed towards a member of their own ethnic group.

Body-related visual information also provides *semantic information* that can be used to perceive another person as similar to or dissimilar from oneself. Such semantic categorizations of self and other include several domains: socio-political affiliation can be a critical factor in such judgements. In Experiment 2, we studied whether this factor influences visuo-tactile remapping. Ten democratic and 10 conservative participants, all politically active, were asked to respond to tactile stimuli on their faces while observing pictures of a face being touched. The faces depicted were of well-known political leaders belonging either to a democratic or to a conservative party (see Methods). By counterbalancing their political positions, observers viewed touch on the bodies of others who were either similar or dissimilar to themselves in socio-political view. If such a political dimension is used to determine similarity between the representation of one's own and others' bodies, then visuo-tactile facilitation should be greater when people observe touch directed towards a member of their own political group. To study how visual information about touch modulates tactile perceptual thresholds, we used the paradigm developed by Serino et al. [12], who created a new version of a tactile confrontation task normally used in brain damaged patients. Subjects were electrically stimulated either on their right, left, or both cheeks and were requested to report the side, or sides, of stimulation. Patients suffering extinction are unaware of the contralesional stimulus in conditions of double stimulation, because of competition between the representations of the two hemispaces [16]. To simulate extinction in healthy participants, we titrated the tactile stimulus intensity to be stronger on one cheek than on the other. We predicted the stronger stimulus would occasionally extinguish the weaker stimulus during double stimulation.

**Figure 1:**
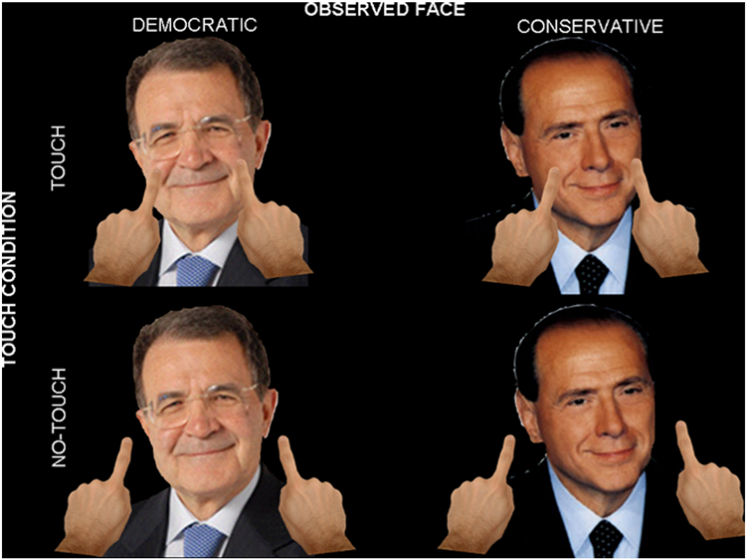
Experimental design. Subjects performed the tactile confrontation task in a two by two design. Over a series of trials they viewed either In-group or Out-group faces, with those groups defined by either ethnic (Experiment 1) or political (Experiment 2) similarity to the viewer. The depicted face could be touched (Touch Condition) or simply approached (No-Touch condition) by one or two fingers on either the right, left or both sides of the depicted face. Figure 1 shows examples of stimuli from Experiment 2: the face of a Democratic (on the left) and a Conservative (on the right) political leader, was either touched (on the top) or simply approached (on the bottom) by two fingers. The pictures on the left represent an In-group condition for a Democratic observer and an Out-group condition for a Conservative observer and vice-versa for the pictures on the right.

During the task, subjects watched a movie showing either an In-group or an Out-group face. In Experiment 1 these groups were defined ethnically, and in Experiment 2 they were defined politically. Over a series of trials, the face depicted was either touched (Touch condition) or simply approached (No-touch condition) by human fingers, on the right, the left, or on both sides. At the zenith of the visual motion in the movie, subjects were given a electro-tactile stimulation on either their right, left, or both cheeks (see Figure 1).

Subjects were instructed to ignore the side of the visual information, and to press a button with the hand corresponding to the side of the tactile stimulus on their own face. An improvement in detecting bilateral tactile stimuli modulated by visual information was taken as an index of visuo-tactile re-mapping. If the re-mapping mechanism is sensitive to the identity of the observed face, a stronger effect should be observed when people view touch directed towards a member of their own ethnic (Caucasian and Maghrebian) or political (Democratic and Conservative) group. Furthermore, if such modulation is a specific effect of viewing touch and not a generic arousal effect of viewing faces, the visual modulation of touch should be expected only when the fingers actually touch the depicted face, and not when they simply approach it.

## Results

### Effects of face similarity on visual remapping of touch

Mean response accuracy to tactile stimuli was computed for each subject in each condition for Experiment 1 and 2. The complete design of the two experiments included 3 types of tactile stimulation (bilateral, left and right), three sides of visual stimulation (bilateral, left and right), two movements direction (touch and no-touch) and two types of image (In-group and Out-group). Half subjects received a stronger tactile stimulation on their left cheek than on their right cheek, and vice-versa the other half. Since no difference was found between these two groups both in Experiment 1 [F(1,12) = .16; p = .69] and in Experiment 2 [F(1, 18) = 1.03; p = .33], we re-coded the sides of stimulation as bilateral, strong and weak stimulation. Two ANOVAs were conducted, one for Experiment 1 and one for Experiment 2, with the within-subjects factors of Image (In-group and Out-group), Touch condition (Touch and No-Touch), Side of tactile stimulation (bilateral, strong and weak) and Side of visual stimulation (bilateral, strong and weak). When necessary, post-hoc comparisons were conducted by means of the Newman-Keuls test.

#### Experiment 1

**Figure 2:**
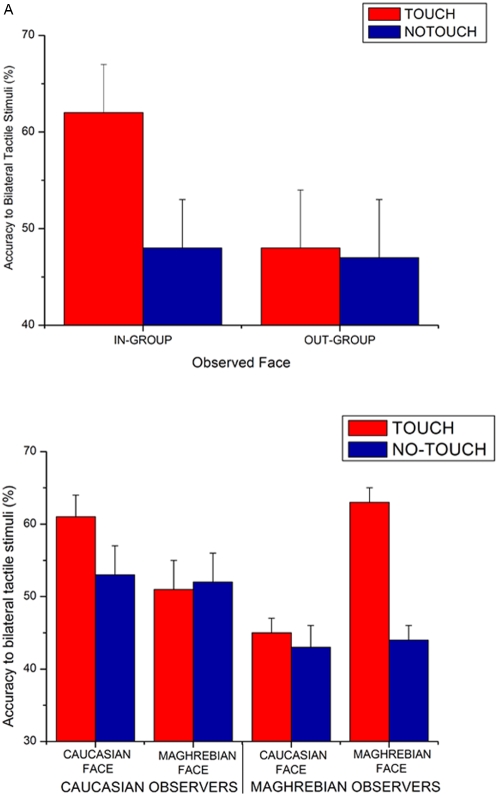
Visual enhancement of touch depends on the Ethnic similarity between the observed and observer's face. Panel A: Tactile perception (measured as accuracy in detecting bilateral tactile stimuli) was enhanced when observers from Caucasian and Maghrebian ethnic groups viewed a face from their own ethnic group (In-group condition; left column) rather than a face from the other ethnic group (Out-group); the effect was specific to the observation of touch (Touch condition, Black bars). Panel B reports data from Caucasian and Maghrebian observers plotted separately.

As far as Experiment 1 is concerned, the main effects of Side of tactile stimulation [F(2,26) = 24.37; p<.00001] and Image [F(1,13) = 5.18; p<.05] were significant. Mean response accuracy to bilateral stimuli was lower (mean = 52%; s.e.m. = 5) than that to weak (mean = 63%; s.e.m. = 4; p<.05) and strong (mean = 95%; s.e.m. = 1; p<.0001) stimuli. Responses to weak and strong stimulation also differ between each other (p<.001). These findings confirm that the present experimental paradigm mimics the extinction phenomenon. Moreover, tactile perception was enhanced by viewing In-group faces (mean accuracy = 73%) in comparison to viewing Out-group faces (67%). This effect needs to be interpreted in the light of the critical three way interaction Image X Touch condition X Side of tactile stimulation [F(2,26) = 6.58; p<.005]. As expected from previous results [12], the detection of bilateral tactile stimuli was modulated by viewing touch. Bilateral trials perception was enhanced when participants observed a face belonging to their own ethnic group being touched (Mean Accuracy for In-group faces = 62%; s.e.m. = 5) in comparison to when they observed the face of a member of the other ethnic group being touched (mean = 48%; s.e.m = 6; p<.0003). This Ethnic Membership effect was specific to the observation of touch, since no difference was found between In-group (mean = 48%; s.e.m = 5) and Out-group faces (mean = 47%; sem = 6) when the fingers merely approached the depicted face (See Fig. 2A). Response accuracy to unilateral trials was instead not modulated by viewing touch or no-touch on the observed faces: these data are reported in Table 1.

**Table 1:** Mean accuracy and s.e.m. for the three types of tactile stimulation when viewing In-group and Out-group faces being touched, or not being touched, in Experiment 1 and Experiment 2.

IMAGE	TOUCH CONDITION	TACTILE STIMULATION	EXPERIMENT 1		EXPERIMENT 2	
			MEAN	S.E.M.	MEAN	S.E.M.
IN GROUP	TOUCH	BILATERAL	62%	5%	60%	3%
		STRONG	96%	1%	88%	3%
		WEAK	68%	6%	62%	5%
	NO TOUCH	BILATERAL	48%	6%	52%	3%
		STRONG	97%	2%	90%	3%
		WEAK	69%	6%	57%	6%
OUTGROUP	TOUCH	BILATERAL	48%	5%	52%	4%
		STRONG	94%	1%	85%	3%
		WEAK	62%	6%	58%	5%
	NO TOUCH	BILATERAL	49%	6%	53%	4%
		STRONG	90%	3%	94%	1%
		WEAK	60%	5%	55%	6%

Even if the effects were analogously present in Caucasian and Maghrebian subjects, for matter of completeness we report the data for bilateral trials detections of the two group of participants plotted separately in Figure 2B.

#### Experiment 2

**Figure 3:**
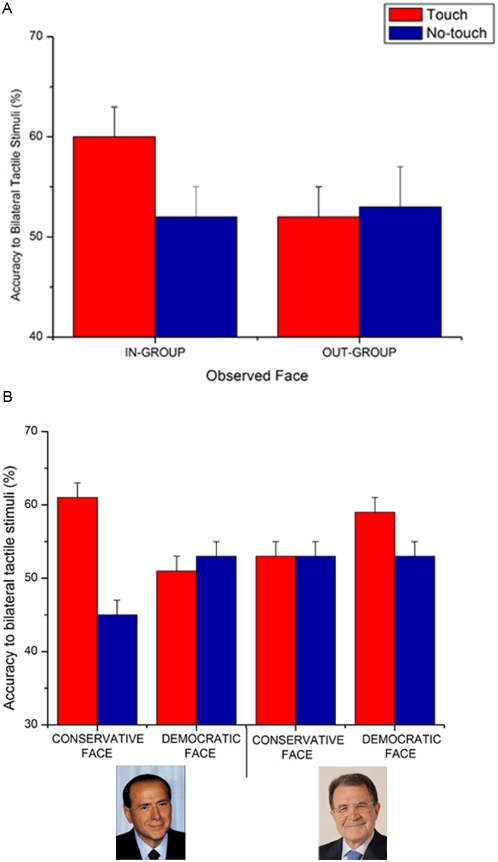
Visual enhancement of touch depends on the Political similarity between the observed and observer's face. Panel A: Tactile perception was enhanced when observers belonging to Democratic and Conservative parties viewed the face of a member of their own political party (In-group) rather than of the opposite (Out-group) political party. Panel B reports data from Conservative and Democratic observers plotted separately.

As far Experiment 2 data are concerned, the main effect of Side of tactile stimulation was significant [F(38,2) = 30.88; p<.00001]. The detection of the strong tactile stimulation (mean accuracy = 89%; s.e.m. = 2) was better than those of the weak (mean = 57%; s.e.m. = 5; p<.0002) and bilateral (mean = 54%; s.e.m. = 3; p<.0002) tactile stimulation. Moreover, the two-way interaction Image by Touch condition was significant [F(1,19) = 9.26; p<.007]. Tactile detection was enhanced when viewing a face of a politician belonging to one's own political group being touched (mean = 70%; s.e.m. = 3) rather than being approached (mean = 65%; s.e.m. = 2; p<.03). This modulation was not found when viewing a face of a politician belonging to the opposite group (touch condition mean accuracy = 65%; s.e.m. = 3; no-touch condition mean accuracy = 67%; s.e.m. = 2; p = .33). The significant effect of this two-way interaction was largely due to the modulation of the detection of bilateral tactile stimuli by visual information. Indeed, the three way interaction Image X Touch X Side of tactile stimulation was significant [F(2,38) = 3.5; p<.05]. Response accuracy to bilateral trials was enhanced when political activists viewed faces of their own party leaders being touched (mean = 60%; s.e.m. = 3) in comparison to when they viewed faces of the opposite party leaders being touched (mean = 52%; s.e.m, = 4; p<.004). The In-group/Out-group difference was not found when the observed face was not touched (In-group: mean = 52%; s.e.m. = 3; Out-group: mean = 53%; s.e.m. = 4) (See Fig.3A). As in Experiment 1, detection of unilateral tactile stimuli was not modulated by viewing touch or no-touch on the observed faces (see Table 1).

Even if the effects were analogously present in Conservative and Democratic subjects, for matter of completeness we report the data for bilateral trials detections of the two group of participants plotted separately in Figure 3B.

### Explicit pleasantness judgements towards the shown faces

The images of faces used during both experiments were chosen to be matched for aesthetic appeal as judged by naïve observers (see Methods). Nevertheless, at the end of both experiments we also asked our participants to judge for pleasantness the faces they had observed. We controlled for pleasantness of the observed faces because other results from our laboratory suggest that viewing more pleasant faces enhanced visual remapping of touch in comparison to viewing less pleasant faces (Giovagnoli, Serino & Làdavas, “I feel what you feel if I like you”, in preparation). To this aim, after they completed the experimental task, we presented subjects with an image of each face and ask them to rate the face for pleasantness on a seven-level scale, from 1, less pleasant to 7, most pleasant. Subjects were explicitly asked: “how much do you like the person in this picture?” The pleasantness rates for images shown in Experiment 1 did not differ when participants judged faces from their own (mean judgements = 3.7) or from a different ethnic group [ 3.6; t(1,13) = .26; p = .62]. In contrast, when at the end of the Experiment 2, the politically active participants were requested to explicitly judge the pleasantness of faces, a significant positive bias was found for faces of leaders belonging to their own party [4.9 as opposed to 2.2; t(1,19) = 44.54; p<.00001]. Thus, although the stimuli used in the two experiments were actually matched for pleasantness - as shown by the naïve judges - political beliefs influenced explicit pleasantness judgments in politically active observers, whereas ethnic membership did not affect explicit judgments towards individual of different ethnic groups.

## Discussion

Viewing touch on the body of others seems to be re-mapped onto one's own somatosensory system, enhancing cortical activity [8]–[10] and perceptual experiences [12]. Such re-mapping increases as a function of the congruity between the observer's and the observed bodies [12]. While previous research has demonstrated that high-order conceptual and social representations of the self and others contribute to judgements of self-other similarity [17], the present findings further demonstrate that such high level representations also modulate the sense of touch. Tactile detection of bilateral stimuli was enhanced when Caucasian observers viewed a Caucasian face compared to when they viewed a Maghrebian face, and the effect was exactly reversed for Maghrebian observers. Importantly, this effect was specifically related to the observation of touch, and was not due to a general arousal effect depending on observing different faces: indeed no-tactile modulation in relationship to ethnic membership was found when participants viewed a face being merely approached by human fingers, but not being touched. Thus ethnic similarity between the self and other bodies modulates visual remapping of touch.

Visual information about the body, besides providing physical information about oneself and co-specifics, also provides *semantic information* that can be used to perceive the other to be similar to versus dissimilar from oneself. Socio-political information is an important factor determining similarity, or dissimilarity, between the identity of self and others. The results of the present study showed that this political factor influences visual remapping of touch. People experience differently a tactile sensation on their own body when they observe the body of another person being touched, if this person has the same or the opposite political view that they have. Detection of sub-threshold tactile stimuli was enhanced when Democratic political activists viewed a face of a Democratic leader, such as Romano Prodi, but not when they viewed a face of a Conservative leader, such as Silvio Berlusconi; the opposite pattern was found for Conservative observers. Again, the effect was specific for the observation of touch, since no modulation of tactile perception was found when the observed face was not touched but only approached by human fingers.

In both experiments, the lack of an effect when the shown face was not touched should be considered, because this excludes a generic effect of arousal or familiarity or attention^1^ related to just seeing similar or dissimilar faces. Moreover, the present results could not be explained on the basis of a perceptual bias, like pleasantness of the shown faces, because the images used in the present experiments were selected so that the faces belonging to different ethnic and political groups were matched for pleasantness (see Methods). Stimuli were chosen from a larger database of pictures which had been judged previously for pleasantness by a sample of 30 naïve judges. Judges were not residents of Italy and did not know the persons depicted in the images. Although the selected faces were equally pleasant, the participants, at the end of the experiment, evaluated the pleasantness of the shown faces differently in the two experiments. The pictures of unknown Caucasian and Maghrebian persons obtained the same pleasantness rates. In contrast, a bias was found in politically active participants: liberal observers judged as more pleasant a face of a liberal leader than that of a conservative leader and vice-versa conservative observers did.

In summary, although the stimuli used in the two experiments were actually matched for pleasantness - as shown by the naïve judges - political beliefs influenced explicit pleasantness judgments towards politicians of different parities in politically active observers, whereas ethnic membership did not affect explicit judgments towards individual of different ethnic groups. Nonetheless, the effects of ethnic and political membership on visual remapping of touch were analogous in the two experiments, showing in this way that visual remapping of touch is a quite automatic process, which is modulated by high-order representations of the self and other, and acts independently by explicit pleasantness judgments towards others.

Potentially, other kinds of information related to the observed faces could have influenced the visual remapping of touch effect. Todorov and colleagues, for instance, identified several positive (e.g. trustworthiness) and negative (e.g. aggressiveness) trait judgments derivable from facial appearance [18]. We did not actually control for all possible information conveyed from viewing a face. However, it is worth noting that 5 different faces for each group were used both for Experiment 1 and Experiment 2. Thus, the effect of any possible bias associated to any given face was balanced by the presence of the other faces. Moreover, preliminarily, we compared subjects' responses to individual faces and we did not find any reliable bias associated to any face. Finally, the fact that we actually found an interaction between the membership of the observers and that of the observed faces weakens the hypothesis of a possible bias in the stimuli, since this bias should have equally affected the results for the two groups of subjects. Thus, even if other factors surely affected the self-other relationship, it is unlikely that these effects could systematically have biased in the present study subjects' responses. Thus, when people observe the face of others, they automatically evaluate different dimensions of faces and these evaluations have important social outcomes [19]. For instance, people automatically categorize a face as belonging to one's own or to a different group: face to face interaction is indeed a crucial aspect of group representation [20]. It is well known that in-group versus out-group categorization influences one's own judgments and behaviours towards others [21].

Recent evidence suggests that in-group out-group categorization modulates automatic activation of approach or avoidance behaviours toward others [22], [23]. This mechanism might have a great impact for survival and therefore might have been selected through evolution [24]: the human species has evolved relying on cooperation between individuals from small, strongly interconnected group [25], most of the time in competition with members of different groups. Results from the present study suggest that this basic mechanism of categorizations developed through evolution has been embodied also into basic mechanisms of multisensory integration, such as visual remapping of touch. The effect described in the present work might be seen as a simple, primitive form of empathy towards the other. For this reason, visual remapping of touch resembles other form of empathy towards the others, such as empathy for pain [26]–[30], which is also modulated by high-order factors, such as individual personality traits [30], personal evaluation of others [26], attention and cognitive appraisal [29]. Future studies might contribute to elucidate similarities and differences between these two forms of “empathy”, also in terms of the underlying neural mechanisms.

In summary, observing touch on a body induces a remapping of tactile input onto the observer's tactile system, resulting in an enhanced ability to perceive a tactile stimulus. The amount of enhancement depends on the similarity between the body of the observer's and that of the observed: the effect is maximum for observing one's own body; when observing the body of others, the effect is stronger as much as the other body is perceived as similar to the self. Similarity is defined both in terms of physical features of the body, but also on the basis of more abstract, conceptual representations of others in relationship to the self.

The neural mechanism underlying this effect is not yet totally clear. Neuroimaging studies show that observing touch modulates the activity of primary [9] or secondary somatosensory [8], [10] regions. Thus, the exact role of SI and SII in this effect is not yet totally clear; future neuroimaging studies should address this issue, which was not directly investigated in the present behavioural study. However, independently from whether SI or SII was the area activated by visual information, any visually-dependent modulation of somatosensory activity might potentially boost tactile perception, in that a pre-activated somatosensory system might become more sensitive to perceive subthreshold tactile stimuli. In the present study, we show that tactile perception, as measured with our tactile confrontation paradigm, is modulated by quite elaborated levels of visual processing, such as the ethnic appearance or political membership, of a shown face. Such complex analysis of visual information cannot be computed within somatosensory cortices, but it is probably computed in high-order visual and associative cortices. For instance, Uddin and colleagues [31]–[32] showed that the neural activity in parietal (inferior parietal lobe) and frontal (inferior frontal gyrus) areas is modulated as a function of viewing one's own face or the face of another person. Furthermore, Mitchell and colleagues identified functionally discrete sub-regions of medio-prefrontal cortex, which differently process information about others as a function of how similar for socio-political views another person is perceived to be to oneself [17], [33]. These prefrontal and parietal regions might be critical to link visual information about a face with the self. The same regions might directly project to somatosensory cortices to modulate visual remapping of touch, since functional and anatomical connections between prefrontal and infero-parietal regions have been extensively demonstrated. In the monkey, several projections have been identified from the inferior parietal lobe [34] and from prefrontal regions [35] to both primary and secondary somatosensory cortices. In humans, converging evidence shows that somatosensory cortices can be modulated by visual information via feedback projections from multisensory areas in parietal and prefrontal regions [36]. Future research will shed light upon the dynamics of the neural mechanism underlying this effect.

## Methods

### Participants

Fourteen healthy subjects were studied in Experiment 1. Seven subjects (4 females) were ethnically Caucasian and 7 were ethnically Maghrebian (3 females). The two groups were matched for age (mean age 24 and 23 years old, respectively) and education (20 and 18 years of schooling). Maghrebian subjects were immigrants from Morocco to Italy, and had been resident in Italy for at least 4 years at the time of the experiment. Twenty healthy subjects, all males, participated in Experiment 2. Ten subjects were recruited at the local headquarters of Italian conservative parties and 10 from Italian democratic parties. All of them were political activists and had been card-carrying party members for at least 3 years.

To be sure about their political affiliations, some days before running the experiment, we checked the correspondence between subjects' political beliefs and the political programs of their favourite parties by using a computer administered questionnaire (www.voisietequi.it) This program presented the subject with 25 statements, each concerning a “hot” political issue, and asked the subject to rate his/her agreement on a seven level scale (0 = completely agree; 7 = completely disagree). A typical example statement was: *“Do you agree to forbid all types of drug use – even marijuana and hashish - and punish drug-addicts with sanctions, such as the suspension of driving-licenses, passports, visas, or jail?”*. The responses given by the subject to each statement were used to calculate a global index of political belief. This index was compared to the one calculated on the basis of the official responses that each party gave to the same statements. In this way, the program computed the distance between the subject's beliefs and the official position of each political party. We compared the distance between democratic and conservative subjects' beliefs and the position of their favourite party. A paired sample t-test [t = 16.64; p<.00001] confirmed that our political activists were closer to the position of their favourite party (mean index distance = 8.5) and distant from the position of the opposite party (17.2).

### Stimuli and apparatus

#### Tactile stimuli

For both experiments, tactile stimuli were delivered by 2 constant current electrical stimulators (DS7A, Digitimer), via 2 couples of neurological electrodes (Neuroline, AMBU) placed on the subject's right and left cheeks. For half of the subjects, the tactile stimulus on the left cheek was set to be more intense than that on the right cheek, and vice-versa for the other half. Prior to the experiment, in absence of visual information, the intensity of the electrical stimuli for each subject was titrated with a staircase procedure to a threshold detection rate of 90% for the stronger stimulus and 60% for the weaker. As a confirmation of the correct titration of the stimuli, in Experiment 1, accuracy for unilateral strong and unilateral weak stimuli was 93% and 63%, respectively, and in Experiment 2, 89% and 57%, respectively. This stimulus arrangement results in a tendency for subjects to fail to report the weaker stimulus during trials with double stimulation. Mean accuracy for bilateral tactile detection was 52% in Experiment 1 and 54% in Experiment 2. Errors almost always favoured reporting the side of the stronger stimulus: probability of reporting the side of the stronger stimulus in case of errors during bilateral stimulation was 92% in Experiment 1 and 90% in Experiment 2.

#### Visual stimuli

Visual stimuli consisted of a movie, presented on a 17″ computer screen placed in front of the subject, at a distance of ∼60 cm. The movie depicted a face (see below), covering an 18 area of about 10×20 cm. In the movie, two fingers, one on the right and one on the left, presented on the lower part of the screen, moved first towards the centrally presented face and then backwards to their starting position. The target of the finger movement was manipulated in two different conditions: in the Touch condition the fingers actually touched the cheeks of the shown face, while in the No-touch condition the fingers pointed to the face from a distance of about 5 cm. In different trials, either the finger on the right, on the left or both fingers moved. A tactile stimulus was delivered to the subject precisely when the fingers reached their visual target. Subjects were instructed to press a button with the hand corresponding to the side where they felt the tactile stimulus on their face (and to press the button with both hands in the case of double stimulation), ignoring the visual stimulation. A PC running C.I.R.O. (www.cnc.unibo.psice.unibo/ciro ) software was used to control the presentation of the stimuli and record responses. In Experiment 1, the depicted face was either a picture, in frontal perspective, of a Caucasian or a Maghrebian person. One of five different faces per ethnic group could be randomly presented in each trial. The only constraint was that male participants were presented with male faces only, and vice-versa for female participants. This caveat was used to avoid any effect due to gender difference between the observed and observer's face. In Experiment 2, the depicted faces were of five well-known leaders of conservative or democratic Italian parties. Only male leaders were presented, and only male subjects participated in the experiment. To avoid any potential bias due to the aesthetic appeal of the depicted face the stimuli were chosen so that In-group and Out-group faces were matched for pleasantness. To this end, several pictures of faces from anonymous Caucasian and Maghrebian individuals were shown to naïve judges. These judges did not take part in the experiment itself, and were simply requested to evaluate the pleasantness of each face on a seven level scale (1 = less pleasant; 7 = most pleasant). Mean perceptual judgements did not differ between Italian (mean judgements = 3.6) and Maghrebian (3.5; p = .54) faces used for Experiment 1.

In Experiment 2, it was important to ensure that pleasantness judgements depended strictly on the perceptual features of the faces and not on more general information about the person depicted. To this end, pleasantness judgements were collected on a sample of 25 naïve foreign judges, resident abroad (in Australia, the US and the United Kingdom). These judges were not familiar with Italian politics and did not recognize any Italian politicians from their pictures or were requested to abstain for judging faces they had recognised. An initial sample of 20 political faces was evaluated, and from these we selected stimuli matched on pleasantness between Conservative and Democratic faces. Mean pleasantness judgements were 3.5 for both types of faces used during the experiment (p = .86).

### Procedure and design

Each experiment consisted of 4 blocks of the tactile confrontation task. In each block, stimuli comprised a factorial combination of the two types of tactile stimulation (unilateral left or right, and bilateral), the two targets of visual stimulation (unilateral left or right, and bilateral), the two fingers movement targets (touch, no-touch) and the two types of faces (In-group or Out-group; 5 Caucasian and 5 Maghrebian faces in Experiment 1; 5 Conservative and 5 Democratic faces in Experiment 2). Each combination was repeated 4 times, for a total of 160 trials per block, presented in random order. Each trial lasted about 3 seconds. Tactile thresholds were checked and re-titrated in absence of visual information before each block.

### Note

1. The different results for Touch and No-touch condition could be potentially explained by an effect of attention, being directed towards the stimulated body part in the Touch, but not in the No-touch condition. However, an attentional factor should equally affect tactile perception when viewing In-group and Out-group faces. Thus, an attentional explanation cannot account for the main finding of the present study, that is the effect of ethnic and political similarity.
